# Serum N-Terminal Pro-B-Type Natriuretic Peptide as a Biomarker of Critical Pulmonary Stenosis in Neonates

**DOI:** 10.3389/fped.2021.788715

**Published:** 2022-01-05

**Authors:** Zhiwei Lin, Yanru Chen, Lin Zhou, Sun Chen, Hongping Xia

**Affiliations:** ^1^Department of Neonatology, Xinhua Hospital, Shanghai Jiao Tong University School of Medicine, Shanghai, China; ^2^Pediatric Cardiac Center, Xinhua Hospital, Shanghai Jiao Tong University School of Medicine, Shanghai, China

**Keywords:** pulmonary stenosis, critical pulmonary stenosis, N-terminal Pro-B-type natriuretic peptide, newborns, biomarker

## Abstract

**Objectives:** To determine the efficacy of serum N-terminal pro-B-type natriuretic peptide (NT-proBNP) levels in predicting critical pulmonary stenosis (CPS) in neonates.

**Methods:** All neonates with pulmonary stenosis (PS) admitted to the neonatal intensive care unit of Xinhua Hospital from October 2014 to December 2020 were retrospectively reviewed. Infants with serum NT-proBNP levels measured within 48 h after birth were enrolled and divided into CPS and non-CPS groups. Serum NT-proBNP levels and cardiac Doppler indices were compared between the two groups. Correlations were determined using the Spearman's rank correlation coefficient. Receiver operator characteristic curve analysis was used to explore the predictive value of NT-proBNP for identifying neonatal CPS.

**Results:** Among 96 infants diagnosed with PS by echocardiography, 46 were enrolled (21 and 25 in the non-CPS and CPS groups, respectively). Serum NT-proBNP levels were significantly higher in the CPS group than in the non-CPS group [3,600 (2,040–8,251) vs. 1,280 (953–2,386) pg/ml, *P* = 0.003]. Spearman's analysis suggested a positive correlation between Ln(NT-proBNP) levels and the transvalvular pulmonary gradient (*r* = 0.311, *P* = 0.038), as well as between Ln(NT-proBNP) levels and pulmonary artery velocity (*r* = 0.308, *P* = 0.040). Receiver operating characteristic curve analysis showed that a cutoff serum NT-proBNP level of 2,395 pg/ml yielded a 66.7 and 78.9% sensitivity and specificity for identifying CPS, respectively. The area under the curve was 0.784 (95% CI, 0.637–0.931). A positive correlation was found between Ln(NT-proBNP) and length of hospital stay (*r* = 0.312, *P* < 0.05).

**Conclusion:** Serum NT-proBNP level was positively correlated with PS severity and could be used as a biomarker to identify CPS in neonates.

## Introduction

Pulmonary stenosis (PS) is a common congenital heart disease characterized by obstruction of the right ventricular outflow tract, accounting for ~8% of all congenital heart diseases (1). Critical PS (CPS) is a life-threatening condition in neonates because of inadequate antegrade pulmonary flow through the right ventricular outflow tract, and it presents as cyanosis and evidence of patent ductus arteriosus (PDA) dependency, which can lead to death without timely treatment, given that the ductal shunting decreases progressively (2). Prostaglandin E_1_ (PGE_1_) should be administered in time to maintain the open ductus arteriosus, increase the pulmonary artery blood flow, and improve hypoxemia. Percutaneous balloon pulmonary valvuloplasty (PBPV) should be performed immediately after stabilization of the patient's general condition (2, 3). Transthoracic two-dimensional echocardiography and color Doppler echocardiography are clinical standards for detecting PS and quantifying its severity (4). However, to the best of our knowledge, no recognized and definite biochemical indicators have been reported to date to assess the severity of PS in neonates. Therefore, a widely feasible biomarker would greatly benefit the promotion of early diagnosis and treatment of CPS, especially in situations wherein bedside echocardiography is not immediately available.

B-type natriuretic peptide (BNP) is a cardiac hormone that is mainly synthesized and secreted by ventricular cardiomyocytes in response to pressure or volume overload. Increases in ventricular wall tension and ventricular load are the main regulatory factors of BNP synthesis and release (5). N-terminal pro-B natriuretic peptide (NT-proBNP) is an inactive by-product, which has a longer half-life in circulation and is more stable in serum. At present, NT-proBNP is relevant to various neonatal diseases, such as pulmonary hypertension (6, 7), PDA (8–14), valular diseases (15), respiratory distress syndrome (16), bronchopulmonary dysplasia (BPD) (7, 17, 18), and retinopathy of prematurity (19, 20). However, limited information is available on the clinical value of NT-proBNP in neonatal PS. We hypothesized that serum NT-proBNP levels were positively correlated with PS severity and could help identify neonatal CPS.

## Patients and Methods

### Patients

All newborn infants with PS admitted to the neonatal intensive care unit of Xinhua Hospital Shanghai Jiao Tong University School of Medicine between October 2014 and December 2020 were retrospectively reviewed. All neonates were diagnosed with PS by echocardiography, and serum NT-proBNP levels were measured shortly after birth. The inclusion criteria were as follows: diagnosis of PS and intact ventricular septum, serum NT-proBNP levels measured within 48 h after birth, and gestational age over 34 weeks. The exclusion criteria were as follows: PS and ventricular septal defect (VSD), pulmonary atresia (PA), other diagnosed complex congenital heart diseases, and severe diseases of other systems. Based on the inclusion and exclusion criteria, the enrolled infants were further divided into the CPS and non-CPS groups. CPS was defined as neonatal PS with cyanosis and evidence of PDA dependency (2).

### Methods

Clinical data were retrieved from the electronic medical record system, including information on clinical management and PBPV treatment. Serum NT-proBNP, troponin I, and CK-MB levels were measured by chemiluminescent immunoassay using the Access 2 Immunoassay System (Beckman Coulter, Inc., USA). Measurements were completed immediately after blood collection and centrifugation. Bedside echocardiography was performed using an ultrasonographic unit with a 5–10 MHz probe. Pulmonary artery velocity (PAV, m/s) was measured, and the transvalvular pulmonary gradient (TVG, mmHg) was calculated using the modified Bernoulli equation (TVG = 4 × PAVmax2). Right ventricular systolic pressure, pulmonary systolic pressure, and peak-to-peak gradients were examined in neonates undergoing PBPV.

### Statistical Analysis

Data analysis was performed using SPSS (version 23.0; IBM, Inc., Chicago, IL, USA). Continuous variables are presented as means ± standard deviations (SD), whereas for non-normally distributed datasets, the median and interquartile range (IQR, 25th and 75th percentile) were used. The proportions were presented as percentages. The *t*-test was used to analyze normally distributed variables. Non-normally distributed data were compared using the rank-sum test. The enumeration data were expressed as *n* (%), and comparisons between groups were performed using the chi-square test or Fisher's exact probability method. The Spearman correlation coefficient was applied to compare Ln(NT-proBNP) with TVG and PAV. Receiver operating characteristic (ROC) analysis was performed to predict the level of serum NT-proBNP that provided the best combination of sensitivity and specificity for identifying CPS. Statistical significance was set at *P* < 0.05.

## Results

**Figure 1:**
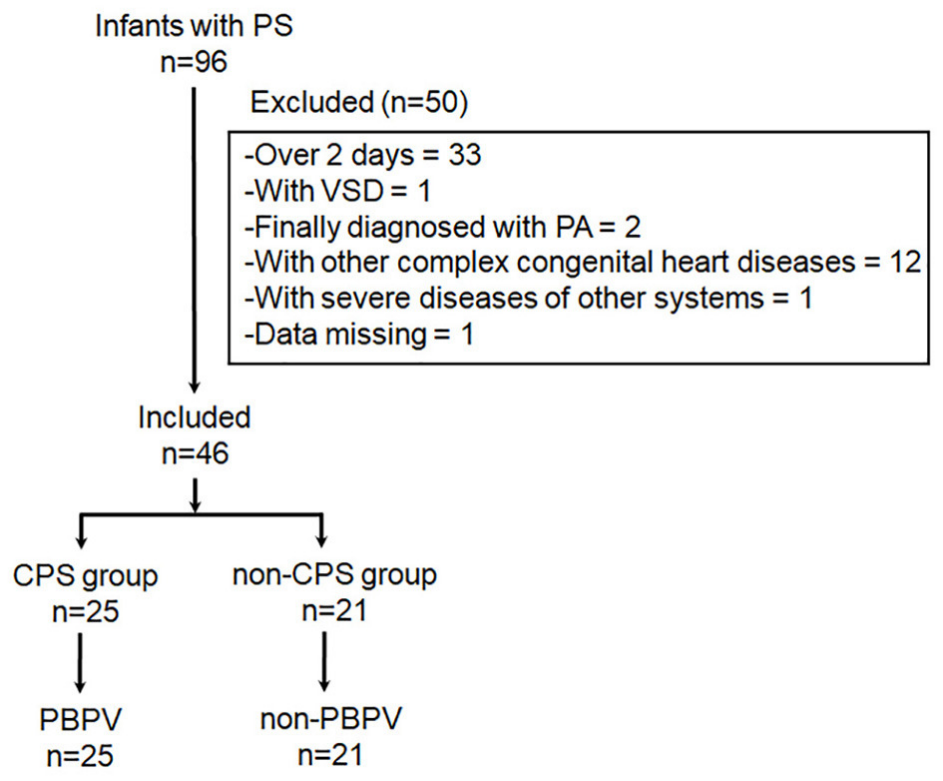
Patient enrollment flowchart.

Among 96 newborn infants diagnosed with PS by echocardiography from October 2014 to December 2020 at Xinhua Hospital, 33 had serum NT-proBNP levels measured over 48 h after birth, the remaining 63 patients had a gestational age over 34 weeks, 1 had PS and VSD, 2 had PA, 11 had other complex congenital heart diseases (1 with right ventricular diverticulum, 4 with double outlet right ventricle, 3 with complete transposition of the great artery, 1 with single ventricle, 1 with aortic stenosis, and 2 with severe TR), 1 had severe pleural effusion and ascites, and 1 had missing data. Overall, 46 newborns were included in our study, with 25 and 21 in the CPS and non-CPS groups, respectively (Figure 1).

The basic clinical characteristics of the two groups are summarized in Table 1. There were no differences in gestational age, birth weight, sex, diameter of the pulmonary ring, and age when serum NT-proBNP levels were measured. The rate of cesarean births was higher in the CPS group than in the non-CPS group. Two infants had serum NT-proBNP levels measured on the second day of life in the CPS and non-CPS groups, respectively, and all the remaining infants had serum NT-proBNP levels measured within the first 24 h after birth. One- and five-minute Apgar scores and percutaneous pulse oxygen saturation (SpO_2_) were significantly lower in the CPS group than in the non-CPS group (*P* < 0.05).

**Table 1:** Clinical characteristics of infants in the CPS and non-CPS groups.

**Characteristics**	**Non-CPS *n* = 21**	**CPS *n* = 25**	***P*-value**
GA, w, mean ± SD	38.90 ± 1.76	38.38 ± 1.78	0.324
BW, g, mean ± SD	3,237 ± 718	3,179 ± 613	0.770
Male, *n* (%)	7 (33.8%)	11 (44.0%)	0.465
Cesarean delivery, *n* (%)	9 (42.9%)	18 (72.0%)	0.048
1-min Apgar score, median (IQR)	10 (9.10)	9 (9.9)	0.000
5-min Apgar score, median (IQR)	10 (9.10)	9 (9.10)	0.043
Postnatal age of testing, days, median (IQR)	0 (0.0)	0 (0.0)	0.661
SpO_2_ %, mean ± SD	93.90 ± 2.64	88.84 ± 5.73	0.000
**Echocardiography**
Diameter of pulmonary ring (mm), mean ± SD	7.38 ± 1.15	7.66 ± 1.23	0.455
PAV max, m/s, mean ± SD	2.99 ± 1.09	4.57 ± 0.57	0.000
TVG, mmHg, median (IQR)	44.00 (20.65, 60.15)	84.00 (70.53, 100.00)	0.000
**Biomarker**
Troponin I, ng/ml, median (IQR)	0.023 (0.012, 0.036)	0.032 (0.019, 0.056)	0.064
CK-MB, ng/ml, median (IQR)	14.9 (8.8, 22.7)	10.3 (7.35, 15.2)	0.247
NT-proBNP, pg/ml, median (IQR)	1,280 (953,2,386)	3,600 (2,040,8,251)	0.003
Ln(NT-proBNP), mean ± SD	7.36 ± 0.77	8.29 ± 0.91	0.000
PBPV, *n* (%)	0 (0%)	25 (100%)	0.000
PGE_1_, *n* (%)	5 (23.8%)	25 (100%)	0.000
Length of hospital stay, days, median (IQR)	6.0 (3.5, 8.0)	12.0 (9.0, 18.0)	0.001

**Figure 2:**
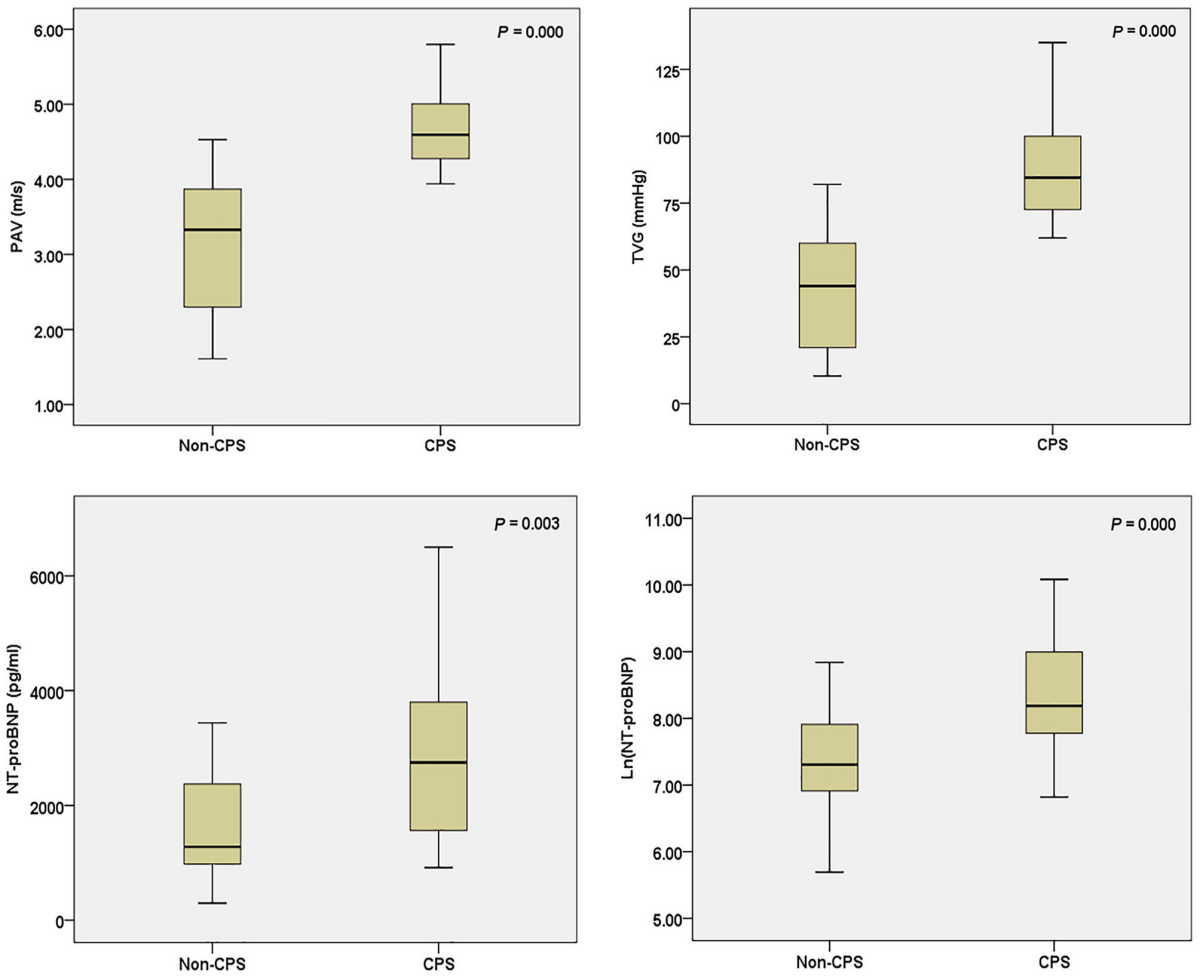
PAV, TVG, NT-proBNP, and Ln(NT-proBNP) in the CPS and non-PS groups.

The PAV and TVG were significantly higher in the CPS group than in the non-CPS group (*P* < 0.01; Figure 2). All patients in the CPS group were treated with PGE_1_. Five (23.8%) patients in the non-CPS group were treated with PGE_1_ shortly after birth, and PGE1 was discontinued after assessment of severity by echocardiography. All infants in the CPS group received PBPV therapy within the neonatal period, whereas none in the non-CPS group received PBPV therapy (*P* < 0.01).

**Figure 3:**
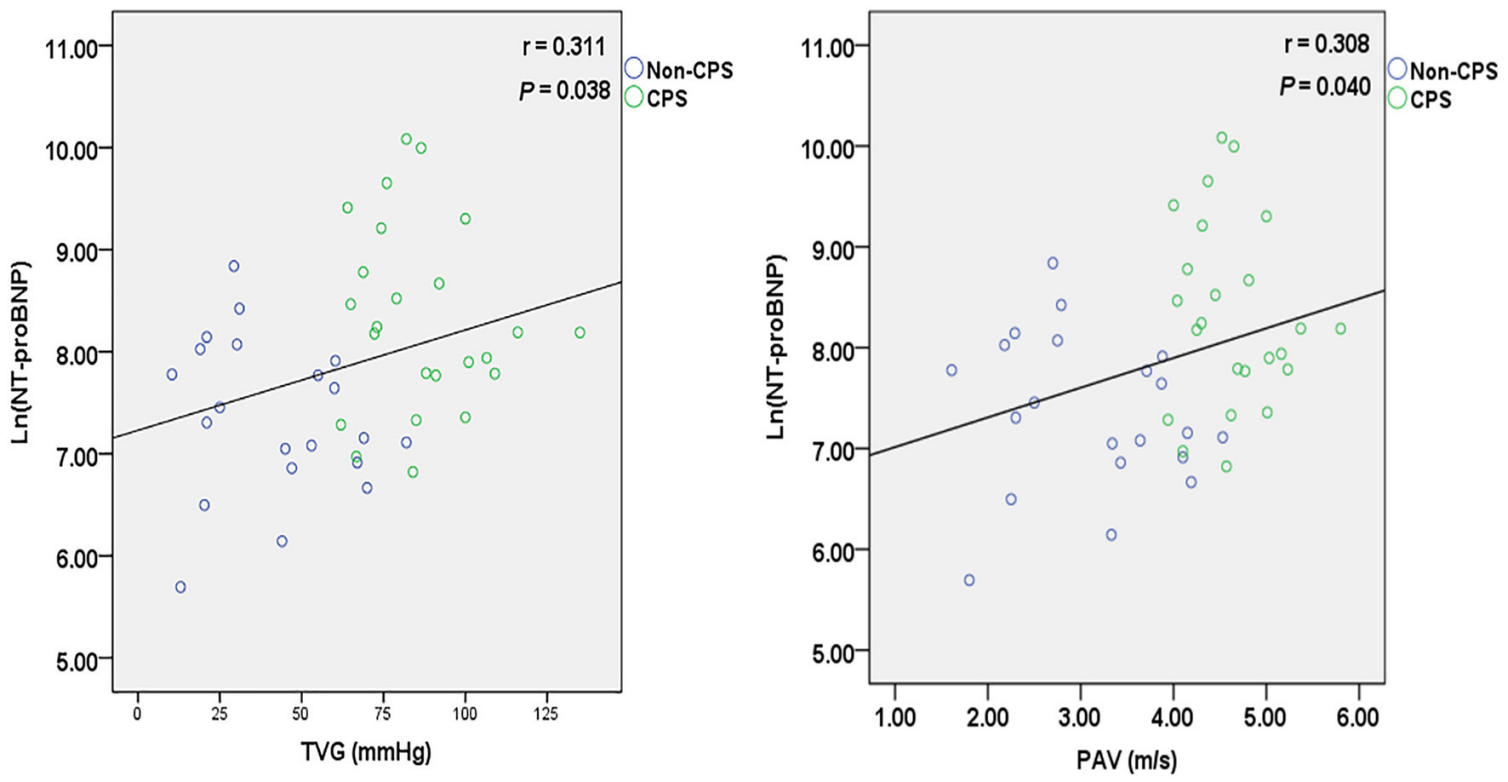
Correlation between Ln(NT-proBNP) level and TVG (*r* = 0.311, *P* = 0.038)/PAV(*r* = 0.308, *P* = 0.040).

**Figure 4:**
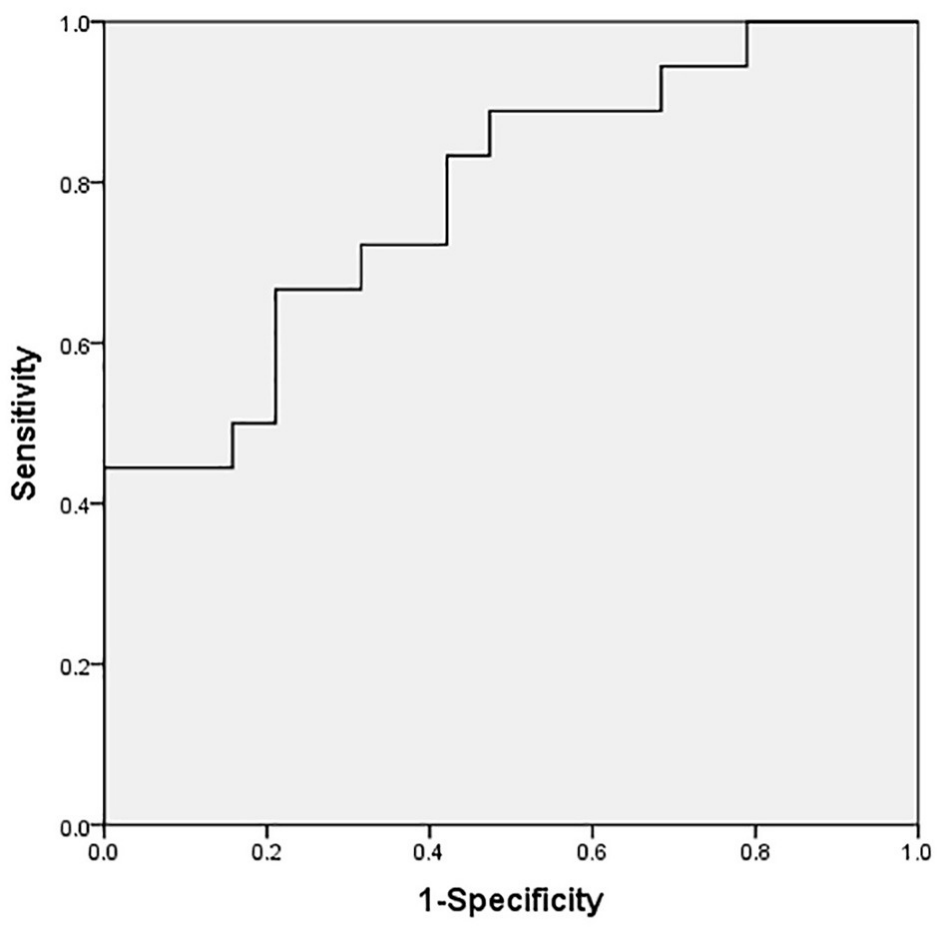
ROC curve of serum NT-proBNP level to predict CPS (cutoff value: 2,395 pg/ml), with a desirable sensitivity (66.7%) and specificity (78.9%) for predicting CPS. The AUC was 0.784 (95% CI, 0.637–0.931).

Serum NT-proBNP levels were significantly higher in the CPS group than in the non-CPS group [3,600 (2,040–8,251) vs. 1,280 (953–2,386) pg/ml, *P* < 0.01] (Figure 2). There were no significant differences in serum troponin I and CK-MB levels between the two groups (*P* > 0.05; Table 1). Spearman's analysis suggested a positive correlation between Ln(NT-proBNP) and TVG (*r* = 0.311, *P* < 0.05) and PAV (*r* = 0.308, *P* < 0.05) (Figure 3). The ROC curve showed that a serum NT-proBNP level of 2,395 pg/ml yielded a 66.7 and 78.9% sensitivity and specificity for detecting neonatal CPS, respectively, with an AUC of 0.784 (95% CI, 0.637–0.931) (Figure 4).

**Figure 5:**
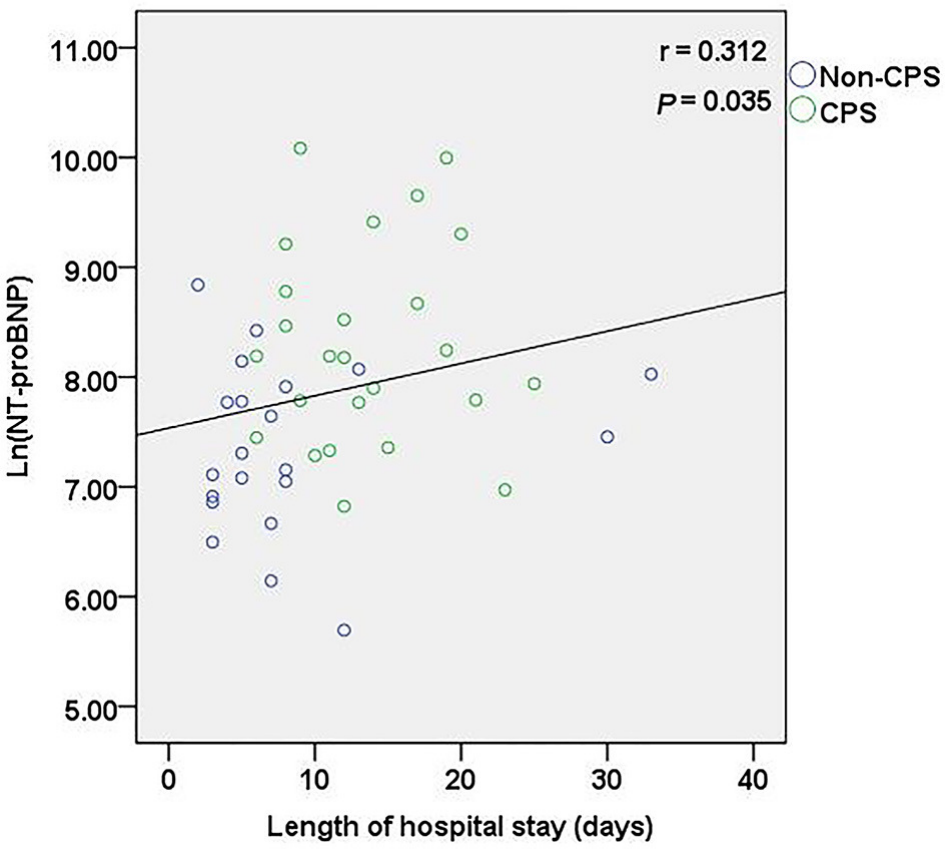
Correlation between Ln(NT-proBNP) and length of hospital stay.

After PBPV therapy, all infants in the CPS group underwent post-operative echocardiography. The alleviation of the obstruction in the right ventricular outflow tract resulted in a significant decrease in the post-operative values for PAV, TVG, and tricuspid transvalvular pressure gradient (*P* < 0.01; Table 2). All infants were discharged from the hospital. The infants in the CPS group had significantly longer hospital days than the non-CPS group [12.0 (9.0–18.0) vs. 6.0 (3.5–8.0) days, *P* < 0.01] (Table 1). A positive correlation was observed between serum Ln(NT-proBNP) and length of hospital stay (*r* = 0.312, *P* < 0.05) (Figure 5). Furthermore, two patients in the CPS group had a second PBPV therapy at 3 and 13 months of age, respectively.

**Table 2:** Pre-operative and post-operative echocardiography in the CPS group.

**Echocardiography**	**Pre-operative *n* = 25**	**Post-operative *n* = 25**	***P*-value**
PAV max, m/s, mean ± SD	4.57 ± 0.57	2.24 ± 0.41	0.000
TVG, mmHg, median (IQR)	84.00 (70.53, 100.00)	20.18 (14.80, 27.00)	0.000
Tricuspid regurgitation velocity, m/s, mean ± SD	5.40 ± 0.87	3.11 ± 0.89	0.000
Tricuspid transvalvular pressure gradient, mmHg, median (IQR)	128.00 (95.50, 139.00)	38.50 (24.60, 54.00)	0.000

## Discussion

This study demonstrated that serum NT-proBNP levels were positively correlated with PS severity and length of hospital stay. In addition, serum NT-proBNP could be used as a biomarker to identify CPS in neonates. In the cases in which bedside color Doppler echocardiography cannot be immediately completed, serum NT-proBNP can be used to determine whether an immediate transfer to a larger center is necessary or prostaglandin administration can be initiated at the place of birth. BNP is primarily synthesized and secreted from ventricular cardiomyocytes in response to ventricular pressure and volume overload (5). NT-proBNP, the inactive split product of pro-BNP, is a well-known biomarker of cardiovascular disease, and its level may increase in a variety of neonatal diseases, and BNP synthesis and secretion are closely related to ventricular pressure overload (21, 22). Oosterhof et al. (21) found that serum BNP levels were associated with right ventricular volume overload in elderly patients with congenital heart disease.

Elevated NT-proBNP and BNP levels have also been found in some premature diseases. A strong positive correlation exists between urinary and plasma NT-proBNP levels (11, 12, 14). In a cohort study of 139 infants with a gestational age of <32 weeks, the median NT-proBNP levels increased by 108% for every 1-mm increase in PDA diameter (10). In a prospective observational study, urinary NT-proBNP levels were positively correlated with ductal diameter and decreased significantly when the PDA closed completely following medical treatment in preterm infants (14). In extremely preterm infants aged 24–48 h with PDA > 1.5 mm, serum NT-proBNP concentrations could reliably predict the development of BPD or death, with the highest diagnostic value at 8-9 days (18). Serum BNP levels have also been reported to be positively correlated with BPD-associated pulmonary hypertension at 36-week corrected gestational age in extremely preterm infants (7). We previously found that in 147 preterm infants with a gestational age of <32 weeks, the serum NT-proBNP levels measured shortly after birth were significantly higher in preterm infants who later developed moderate/severe BPD or died (17).

To date, only few studies have reported biomarkers for the severity of PS in neonates. Wang et al. (23) found a significantly negative correlation between Elabela plasma concentration and TVG in children aged 0–3 years with PS or PA with intact ventricular septum. In a study of 31 children with a mean age of 4.77 years with right ventricular overload due to different types of congenital cardiac diseases, plasma BNP concentrations were positively correlated with right ventricular pressure and volume overload (24). In dog experiments, the plasma concentration of NT-proBNP was positively correlated with the severity of PS (25). El Tahlawi et al. (26) found that serum troponin I correlated positively with PS severity and was associated with right ventricular dysfunction in 50 patients with a mean age of 4.35 years. However, in our study, although there was a slight increase in serum troponin I levels in the CPS group, there was no significant difference between the two groups, possibly because the neonates enrolled in our study were diagnosed with PS shortly after birth. In elderly children with PS, long-term pressure overload may cause functional changes and injury of cardiomyocytes of the right ventricle, leading to significantly elevated troponin I levels in circulation (27).

To the best of our knowledge, this is the first study to investigate serum NT-proBNP levels in neonates with PS. In neonatal CPS, early identification, timely PBPV, or surgical treatment is warranted. Serum NT-proBNP is a useful, safe, and non-invasive diagnostic biomarker to help identify CPS in newborn infants in clinical practice, especially when bedside color Doppler echocardiography cannot be completed promptly.

### Limitations

The limitations of our study are as follows: (1) It was a single-center retrospective study with a relatively small sample size. (2) We excluded neonates with gestational age <34 weeks and neonates without serum NT-proBNP levels measured within 48 h after birth because the NT-proBNP level was found to be associated with gestational age and postnatal age. Nevertheless, these factors may still have some effects on serum NT-proBNP levels. (3) Serum NT-proBNP is not a specific biomarker for CPS and thus, cannot be utilized to diagnose PS in isolation. Echocardiography is still necessary to diagnose CPS and rule out any other congenital heart diseases.

## Conclusions

Serum NT-proBNP level was positively correlated with TVG and had a predictive value for CPS in neonates. Serum NT-proBNP level could be used as a biomarker to assess the severity of PS in newborn infants.

## Data Availability Statement

The raw data supporting the conclusions of this article will be made available by the authors, without undue reservation.

## Ethics Statement

The studies involving human participants were reviewed and approved by Ethics Committee of Xinhua Hospital Affiliated to Shanghai Jiao Tong University School of Medicine. Written informed consent from the participants' legal guardian/next of kin was not required to participate in this study in accordance with the national legislation and the institutional requirements.

## Author Contributions

HX conceived and designed the study. ZL and YC performed the data analysis and wrote the manuscript. SC provided assistance for data acquisition. ZL and LZ collected the data. All authors reviewed the manuscript.

## Funding

This work was partially supported by a grant from the National Natural Science Foundation of China (81200458 to HX).

## Conflict of Interest

The authors declare that the research was conducted in the absence of any commercial or financial relationships that could be construed as a potential conflict of interest.

## Publisher's Note

All claims expressed in this article are solely those of the authors and do not necessarily represent those of their affiliated organizations, or those of the publisher, the editors and the reviewers. Any product that may be evaluated in this article, or claim that may be made by its manufacturer, is not guaranteed or endorsed by the publisher.
